# The Contribution of Dostarlimab to First-Line Treatment of Advanced or Recurrent Endometrial Cancer in Spain: A Multi-Criteria Decision Analysis Approach

**DOI:** 10.3390/jcm15155808

**Published:** 2026-07-24

**Authors:** Maria-Pilar Barretina-Ginesta, Cristina Martín Lorente, Constanza Maximiano, Raúl Díez, EC-MCDA Group, Asís Ariznavarreta, Ana Calvo, Francesc Soler

**Affiliations:** 1 Medical Oncology Department, Institut Català d’Oncologia (ICO), Girona Biomedical Research Institute (IDIBGI), Department of Medical Sciences, Medical School University of Girona (UdG), 17007 Girona, Spain; 2 Medical Oncology Department, University Hospital Santa Creu i Sant Pau, 08041 Barcelona, Spain; 3 Medical Oncology Department, Puerta de Hierro–Segovia de Arana Health Research Institute (IDIPHISA), University Hospital Puerta de Hierro, 28222 Madrid, Spain; 4 Pharmacy Department, University Hospital of Getafe, 28905 Madrid, Spain; 5 Outcomes’10 (a ProductLife Group Company), 12071 Castellón de la Plana, Spain; 6 Pharmacy Department, Institut Català d’Oncologia (ICO), 08908 Barcelona, Spain

**Keywords:** multi-criteria decision analysis, MCDA, dostarlimab, endometrial cancer, EVIDEM, Spain

## Abstract

**Objectives**: To estimate the added value of dostarlimab compared to durvalumab and pembrolizumab as first-line treatment for adult patients with primary advanced or recurrent endometrial cancer (EC) in Spain using a Multi-Criteria Decision Analysis (MCDA) framework. **Methods**: Following the Evidence and Value: Impact on Decision Making (EVIDEM) framework and International Society for Pharmacoeconomics and Outcomes Research (ISPOR) recommendations, 25 Spanish experts (12 medical oncologists and 13 hospital pharmacists) weighted and scored 11 quantitative and 5 qualitative criteria based on an evidence matrix from a literature review. **Results**: Efficacy and safety were weighted as the most important criteria. Dostarlimab showed positive added value in both comparisons with scores of 0.58 versus durvalumab/durvalumab + olaparib and 0.50 versus pembrolizumab. Dostarlimab and pembrolizumab are the only therapies reimbursed in the Spanish public health system; therefore, their comparative assessment is of clinical and strategic relevance. While pembrolizumab may show longer progression-free survival (PFS) and response duration in specific subgroups, the evidence for dostarlimab—including a statistically significant improvement in overall survival (OS) in the overall population as a primary endpoint, more robust data, and favorable long-term quality-of-life outcomes—was considered by the expert panel to support a more favorable assessment. **Conclusions**: Within the MCDA framework, experts consider dostarlimab an option with a higher value contribution for adult patients with primary advanced or recurrent EC. Its clinical benefits, improved quality-of-life outcomes, and alignment with healthcare priorities promote treatment continuity and adherence in clinical practice. MCDA provided a transparent approach for multidimensional evaluation of these therapies.

## 1. Introduction

Endometrial cancer (EC), which originates in the inner lining of the uterus, is the most common form of uterine cancer and is therefore often referred to as uterine cancer [1]. It represents the most frequently found gynecological malignancy in developed countries, with an incidence that continues to increase due to population aging and the rising prevalence of risk factors, such as obesity and metabolic disorders [2]. According to the most recent data from the 2026 report of the Spanish Society of Medical Oncology (SEOM), uterine cancer ranks as the fourth most common form of cancer among women in Spain, with an estimated number of 7759 new cases projected for 2026 [3]. In terms of mortality, 1716 deaths due to malignant uterine tumors were reported in 2024 [3].

Most EC cases are diagnosed at ages between 65 and 75 years [4]. While approximately 80% of cases are diagnosed at an early stage, up to 20% of patients present with advanced disease or experience recurrence [4], and prognosis in this population remains poor [5].

EC is a clinically and molecularly heterogeneous malignancy influenced by epidemiological, hormonal, metabolic, and genetic factors. Established risk factors include advanced age, certain ethnic backgrounds, elevated body mass index, prolonged estrogen exposure, tamoxifen therapy, early menarche, late menopause, low parity, metabolic syndrome, and familial or inherited genetic predisposition [6]. At the molecular level, The Cancer Genome Atlas (TCGA) identified four major molecular subgroups of EC: POLE-ultramutated, microsatellite instability-hypermutated, copy-number low, and copy-number high tumors [4,7]. In clinical practice, this classification is commonly approximated using surrogate markers, including POLE mutation status, mismatch repair (MMR) status, and p53 expression [4,7]. Defects in the MMR system lead to microsatellite instability (MSI), classified as high (MSI-H) or low (MSI-L) according to the degree of instability in MMR protein-coding genes [8]. Tumors with deficient mismatch repair and high microsatellite instability (dMMR/MSI-H) account for approximately 25–30% of EC cases and are typically associated with an intermediate prognosis [8,9]. In contrast, the majority of tumors exhibit proficient mismatch repair and microsatellite stability (pMMR/MSS), representing roughly 75% of cases [10]. These molecular features have prognostic relevance and increasingly inform therapeutic decision-making. In the advanced or recurrent setting, immune checkpoint inhibitors have become particularly relevant [4,7].

For decades, the combination of carboplatin plus paclitaxel represented the standard first-line systemic therapy for advanced or recurrent EC [11]. However, outcomes were limited, with a median overall survival (OS) of less than three years [5].

The inclusion of immunotherapy in first-line disease management has significantly altered this treatment paradigm [12,13]: phase III clinical trials evaluating immune checkpoint inhibitors in combination with chemotherapy have demonstrated meaningful improvements in clinical outcomes [13]. Accordingly, dostarlimab, durvalumab (as monotherapy for dMMR/MSI-H patients and in combination with olaparib for pMMR/MSS patients), and pembrolizumab combined with chemotherapy have received regulatory approval in Europe for the first-line treatment of adult patients with primary advanced or recurrent EC who are candidates for systemic therapy [14,15,16]. Dostarlimab and pembrolizumab are anti-PD-1 antibodies, durvalumab is an anti-PD-L1 antibody, and olaparib is a PARP inhibitor [17,18,19].

Nevertheless, while these therapeutic advances have improved patient outcomes, they have also increased the complexity of clinical decision-making. Recent international clinical guidelines emphasize that molecular classification should be integrated into the diagnostic and therapeutic pathway of EC, as MMR/MSI status, POLE mutations, and p53 abnormalities have important prognostic and predictive implications and guide treatment selection in advanced or recurrent disease [4]. Healthcare systems face increasing pressure to ensure timely and sustainable access to innovative, high-cost therapy [20]. Furthermore, the rapid incorporation of several immune checkpoint inhibitor-based regimens into first-line therapy has created a scenario in which comparative value assessment should consider not only efficacy and safety, but also biomarker-defined populations, quality of evidence, patient benefit, and health-system sustainability [5,15,16]. In this dynamic environment, Multicriteria Decision Analysis (MCDA) offers a structured and transparent approach [21] which is being increasingly used in real practice to prioritize certain healthcare interventions or assess the value of drugs [22]. MCDA is a widely adopted tool that provides a systematic, multidimensional, and transparent framework for evaluating pharmaceuticals by considering a wide range of factors and their respective contributions to the decision-making process [21].

This study aimed to apply an MCDA approach to assess the value of three first-line treatments in adult patients with advanced or recurrent primary EC who are candidates for systemic therapy within the Spanish context, taking a comprehensive approach. Specifically, we sought to assess the value contribution of dostarlimab compared to durvalumab and pembrolizumab. By providing a structured evaluation, this analysis aims to inform more evidence-based decision-making regarding the use of these three treatments.

## 2. Materials and Methods

### 2.1. Study Design

The MCDA methodology was applied in accordance with the recommendations of the International Society for Pharmacoeconomics and Outcomes Research (ISPOR) [15,20]. The study was designed following the Evidence and Value: Impact on Decision Making (EVIDEM) framework, which promotes transparent and structured healthcare decision-making by integrating multiple value dimensions [16]. The EVIDEM framework comprises 20 criteria, of which 13 are quantitative and 7 are qualitative/contextual. The 13 quantitative criteria are grouped into five domains (CORE Model): need for intervention (three criteria), comparative outcomes of the intervention (three criteria), type of benefit provided by the intervention (two criteria), economic consequences (three criteria), and level of knowledge or evidence regarding the intervention (two criteria). The qualitative/contextual criteria are grouped into three domains: normative criteria, feasibility criteria and opportunity costs. The methodological process comprised four sequential phases: constitution and training of the Scientific Committee, evidence identification and construction of the evidence matrix, individual weighting and scoring by the expert panel, and calculation of the overall value contribution of each treatment comparison. This design was considered appropriate for the study objective because the aim was to assess the multidimensional value contribution of available first-line treatment options, rather than to establish direct comparative clinical effectiveness.

### 2.2. Scientific Committee

A Scientific Committee (SC) was established, composed of five experts appointed based on their recognized expertise in EC management and their active involvement in healthcare decision-making processes. The SC participated in all stages of the project and included three medical oncologists and two hospital pharmacists from five healthcare centers across Spain. Before starting work, the Committee members attended an online meeting to receive training on the methodology. The role of the SC was to review the available evidence, agree on the final set of criteria and sub-criteria, and ensure that the evaluation framework was clinically relevant and applicable to the Spanish healthcare context.

### 2.3. Evidence Matrix

A rapid review was conducted, consisting of a comprehensive, manual, and organized analysis of the published literature [21,22], with the aim of gathering relevant information on the disease, its current management in Spain, and the available evidence on dostarlimab and its comparator drugs. The international database PubMed was searched using Medical Subject Headings (MeSH) and free-text terms combined with the Boolean operators “AND” and “OR” (Supplementary Table S1). In addition, grey literature was reviewed by consulting topic-related scientific journals and the websites of relevant national and regional healthcare societies.

Following the EVIDEM methodology, the first step was to define the evaluation framework for advanced or recurrent EC and the treatments authorized in Europe [10]. Based on the evidence gathered, a preliminary list of potential criteria and sub-criteria was developed for inclusion in the evaluation framework. This list was previously shared with the SC, which reviewed it and selected those considered relevant. The results were then presented at a virtual meeting, during which they were discussed and a consensus was reached. The criteria and sub-criteria ultimately included in the evaluation framework were selected by simple majority. The evidence identified in the literature review was then mapped to the chosen criteria and sub-criteria to construct the evidence matrix, which presented the relevant information in a structured and standardized manner.

The evaluation framework and the corresponding evidence matrix were subsequently included in an online questionnaire for weighting and scoring, respectively. An explanatory video on the MCDA methodology was also provided to help participants understand the framework and the assessment process. The questionnaire was pilot-tested before launch to verify clarity, internal consistency, and usability of the online platform.

#### 2.3.1. Participants

A total of 25 participants, including 20 external experts and the 5 members of the SC, completed the questionnaire. Among them, 12 oncologists and 13 hospital pharmacists (HP), representing 11 of the 17 Spanish autonomous communities (ACs). Experts were selected and invited to participate by the sponsor, based on their expertise in the management of EC. Experts were selected based on their clinical experience in the management of advanced or recurrent EC, their involvement in hospital-based decision-making, and their representation of the two specialties most directly involved in treatment selection and access decisions: medical oncology and hospital pharmacy. Geographic diversity across Spanish autonomous communities was also considered. They were provided with a personalized access password via email, along with a link to the online questionnaire hosted on a website dedicated to the project. Weighting and scoring were performed individually and independently by each expert. Once submitted, individual responses and aggregated results were locked and could not be modified.

All procedures followed were in line with the ethical standards of the Declaration of Helsinki. Given the nature of the study, which did not involve the collection of clinical data from participants nor the evaluation of drugs or interventions in humans, approval from an ethics committee was not required, as defined in Royal Decree 957/2020, of 3 November [23], and in the Memorandum of Cooperation between Ethics Committees [24]. All participants were required to provide informed consent before joining the project; this was mandatory to be able to participate in the study. The questionnaire was launched and made available between 10 October and 20 October 2025.

#### 2.3.2. Criteria Weighting and Scoring

The weighting process makes it possible to determine the relative importance of the criteria and sub-criteria within the evaluation framework. To this end, participants conducted a hierarchical weighting exercise, allocating points to each item out of a total of 100 (with higher scores indicating greater importance), first across the criteria and then across the corresponding sub-criteria. This hierarchical approach ensured that the relative importance of each criterion was captured within the overall structure of the EVIDEM framework and that weights could subsequently be normalized for the calculation of value contribution.

Subsequently, each participant assigned a score to each criterion and sub-criterion in the evidence matrix. For the quantitative criteria, two options were considered. Scores for the absolute criteria or sub-criteria (those not involving comparisons with alternative interventions) ranged from 0 to 5, where 0 represented the lowest value and 5 the highest. In the case of relative criteria or sub-criteria (those involving comparisons with alternative interventions), the scoring scale ranged from −5 to 5 to capture the full spectrum of comparative effects. A positive score indicated that dostarlimab was considered to provide greater value than the comparator for the criterion assessed, whereas a negative score indicated lower value relative to the comparator. A score of 0 indicated no relevant difference or insufficient evidence to support a directional judgement. The comments and reflections that prompted the experts’ scores were compiled in a qualitative manner.

Finally, the contextual (qualitative) criteria encompass factors related to the healthcare system, such as the organizational impact, equity, and acceptability of the intervention. These criteria were not scored quantitatively, but were classified qualitatively as having a negative, neutral, or positive impact. This classification helps to contextualize the value contribution of dostarlimab within the specific framework of the Spanish healthcare system.

#### 2.3.3. Statistical Analysis and Value Contribution Calculation

For each criterion and sub-criterion, the mean, standard deviation (SD), and the minimum and maximum scores were calculated. Based on the scores assigned by the participants and the corresponding weights of the criteria, the total value contribution was calculated for dostarlimab vs. durvalumab/durvalumab + olaparib and dostarlimab vs. pembrolizumab. The value contribution (Vx) of each quantitative criterion was calculated as the product of its normalized weight (Wx; ∑Wx = 1), derived using the hierarchical weighting method, and its standardized score (Sx = score/5). The resulting value contributions were expressed on a scale ranging from −1 to 1, where −1 indicates a negative or zero contribution and 1 indicates a positive contribution. The overall value contribution was obtained by summing the individual value contributions across all quantitative criteria (V = ∑Vx). All statistical analyses were performed using Stata software, version 14 (StataCorp LP, College Station, TX, USA).

Within the EVIDEM framework, subcriteria can be used to provide a more detailed understanding of the main criteria by breaking down complex dimensions of value. However, they are not mandatory for calculating the overall value contribution. In this study, subcriteria were collected to support interpretation and provide a more granular description of treatment differences. To avoid double counting, they were not included in the final value calculation, as their content was already captured within the corresponding main criteria. Therefore, the final value contribution was calculated using only the main quantitative criteria, while subcriteria were used descriptively to contextualize the results.

## 3. Results

Of the 20 decision criteria included in the EVIDEM framework, the expert panel selected 11 of the 13 quantitative criteria and 5 of the 7 qualitative/contextual criteria. Included and excluded criteria are shown in Supplementary Table S2. During the review process, the panel further defined specific sub-criteria within 9 quantitative and 2 qualitative criteria. Furthermore, the literature review conducted to complete the evidence matrix did not identify any published indirect comparisons between dostarlimab and the evaluated comparators, confirming a lack of existing comparative data in this setting.

### 3.1. Weighting of the Criteria

**Figure 1:**
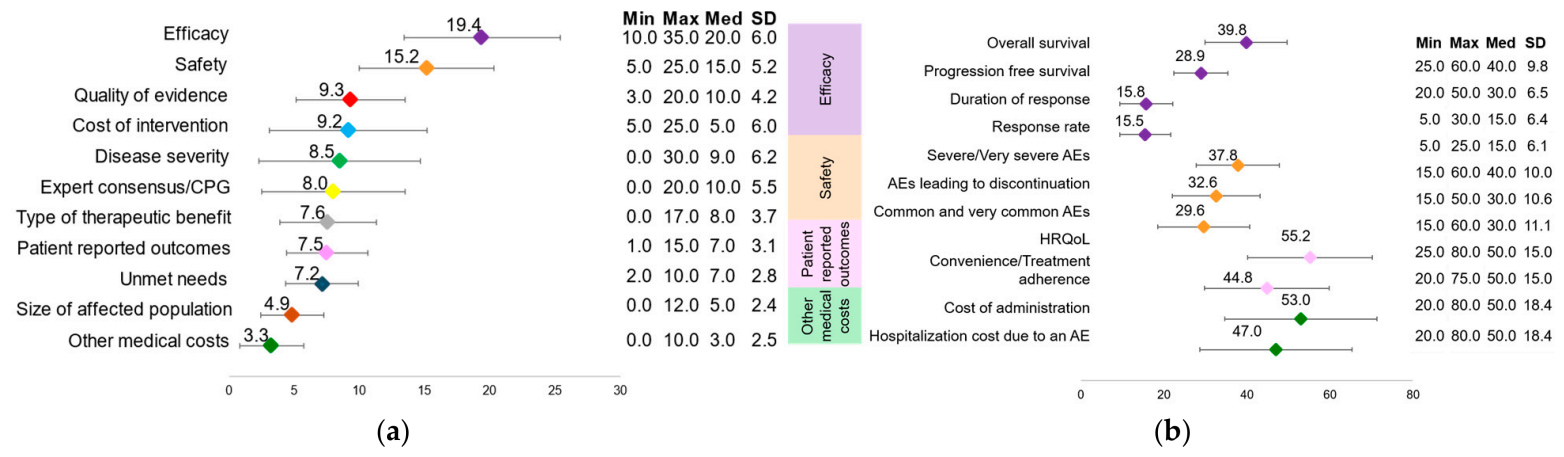
Experts allocated a total of 100 points across the overarching criteria and 100 points within each main criterion among their corresponding sub-criteria. (**a**) Relative importance of the criteria (**a**,**b**) sub-criteria. AE, adverse events; CPG, clinical practice guidelines; HRQoL, health-related quality of life.

As shown in Figure 1, the experts considered the efficacy and safety of treatment to be the most relevant criteria, receiving the highest mean (SD) scores (19.4 [6.0] and 15.2 [5.2], respectively). The remaining criteria were rated more consistently, with mean scores ranging from 7.2 to 9.3 points, except for the two lowest-rated criteria: population size and other medical costs of the treatment, which received mean (SD) scores of 4.9 (2.4) and 3.3 (2.5) points, respectively.

The distribution of the 100 points across the comparative sub-criteria showed a generally balanced allocation of relative importance within each criterion. However, within the efficacy domain, OS was assigned greater importance than the other sub-criteria (Figure 1).

### 3.2. Scoring of the Criteria

The ‘disease severity’ criterion received the highest mean (SD) score (4.5 [0.5]) within the ‘need for intervention’ domain, followed by the ‘unmet needs’ criterion (mean [SD], 4.1 [0.8]). Regarding the comparative criteria, all outcomes were scored at group level in favor of dostarlimab. In comparison with durvalumab/durvalumab + olaparib, the highest mean (SD) scores were 2.9 (1.3) for cost of intervention and 2.2 (1.3) for patient-reported outcomes (PROs). With regard to the comparison with pembrolizumab, the scores were 1.8 (1.6) for efficacy and 1.6 (1.6) for cost of intervention. For the remaining absolute criteria, the ‘Expert consensus/CPG’ received a score of 4.7 (0.6) and the ‘Quality of evidence’ score was 4.2 (0.7) (see Table 1 for the scores assigned to each criterion and comments from the expert panel).

### 3.3. Scoring of Sub-Criteria

**Figure 2:**
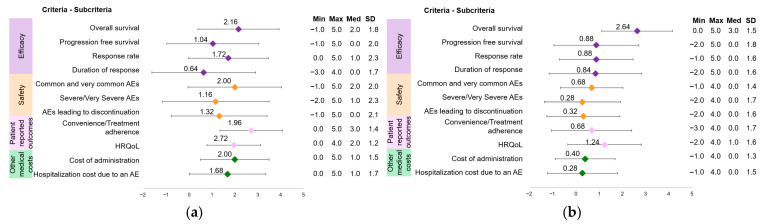
Mean sub-criteria scores. (**a**) Dostarlimab vs. durvalumab/durvalumab + olaparib (**a**,**b**) dostarlimab vs. pembrolizumab. AE, adverse event; HRQoL, health-related quality of life.

The various comparative sub-criteria were scored to gain a more detailed understanding of the parameters that most distinguish the treatments (Figure 2). The sub-criteria were ranked according to the scores obtained in the weighting exercise.

In the comparison group between dostarlimab and durvalumab/durvalumab + olaparib, the greatest differences in efficacy were found in the OS score (mean [SD], (2.16 [1.8]) and in the response rate (1.72 [2.3]). With regard to safety, the greatest difference was found in the sub-criterion of common and very common AEs, with a score of 2.0 (2.0). In terms of PROs sub-criteria, both patient’s quality of life and treatment convenience received high scores (2.72 [1.4] and 1.96 [1.2], respectively). Finally, in relation to other medical costs, dostarlimab was considered less costly compared to durvalumab/durvalumab + olaparib.

In comparison with pembrolizumab, efficacy also favored dostarlimab, particularly in terms of OS (2.64 [1.5]). Other efficacy-related parameters also favored dostarlimab, albeit with smaller differences (around 0.88). With regard to safety, dostarlimab also received favorable scores, with the sub-criterion for common and very common AEs being the highest (0.68 [1.4]). For PROs, the improvement in adherence was scored at 1.24 (1.6), and quality of life at 0.68 (1.7). Lastly, differences in other medical costs were less pronounced, with values ranging from 0.28 (1.5) to 0.40 (1.3).

### 3.4. Contextual Criteria Scoring

The expert panel assessed the extent to which contextual criteria influenced the overall value of the intervention. These criteria were classified into three categories according to their interaction with the healthcare context: those that align with the context and support the intervention (positive impact), those that conflict with the context and hinder its implementation (negative impact), and those that do not clearly exert either a favorable or unfavorable influence. This type of qualitative analysis is inherently complex and, while useful for framing and contextualizing therapeutic strategies, may not fully capture the breadth or nuance of individual expert opinions, and therefore should be interpreted with caution.

**Figure 3:**
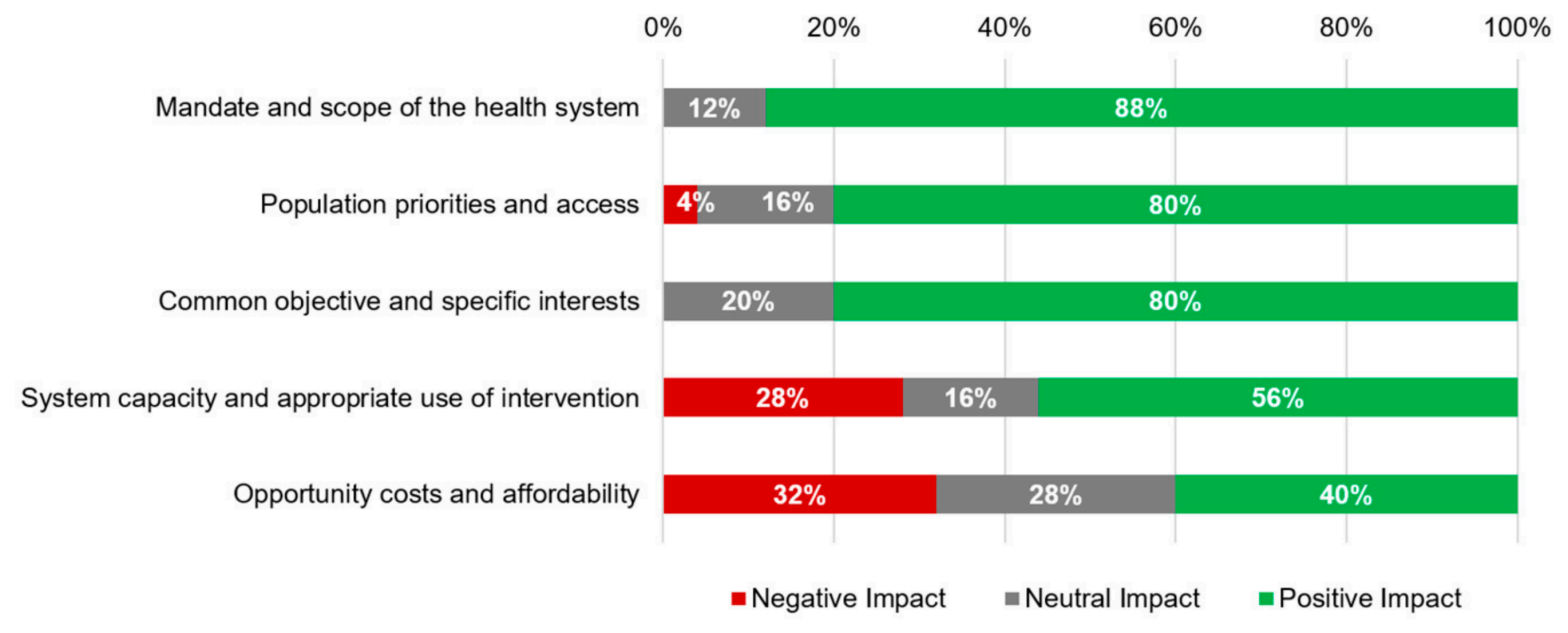
Scores of the contextual criteria.

As illustrated in Figure 3, the contextual criteria were generally perceived as having a positive impact on the value of the intervention. The negative impact reported by 28% of experts in relation to system capacity and intervention use was primarily attributed to the absence of a shared-risk agreement for dostarlimab. This limitation was considered to restrict access when compared with pembrolizumab, which had benefited from such agreements. Conversely, the negative perceptions regarding opportunity costs and affordability reflected concerns among some experts due to the high costs associated with these therapeutic alternatives.

### 3.5. Value Contribution of Dostarlimab

**Figure 4:**
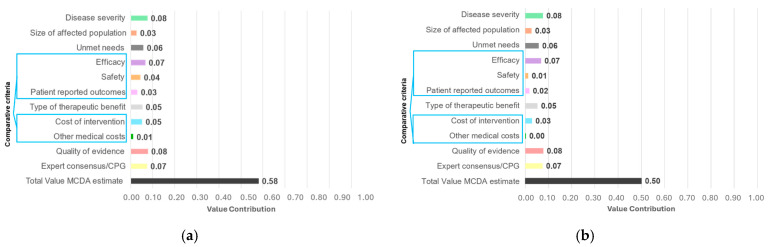
Value contribution of each comparison. (**a**) Dostarlimab vs. durvalumab/durvalumab + olaparib (**b**) and dostarlimab vs. pembrolizumab. CPG, clinical practice guidelines; MCDA, multicriteria decision analysis.

The sum of individual weights and scores assigned by the panel members across all criteria resulted in overall value estimates on a scale from 0 to 1, indicating that dostarlimab provides a positive added value regardless of the comparator. The comparison of dostarlimab versus durvalumab/durvalumab + olaparib resulted in a value of 0.58, while the comparison with pembrolizumab yielded a value of 0.50 (Figure 4). The observed difference of +0.08 reflects greater safety, better PROs, and greater cost-effectiveness compared to durvalumab/durvalumab + olaparib. In terms of treatment efficacy, both comparisons resulted in a similar value contribution of 0.07. Therefore, the total value contribution was positive in both comparisons, with a slightly higher value observed versus durvalumab/durvalumab + olaparib than versus pembrolizumab. This difference was not mainly attributable to efficacy, but rather to other value dimensions, particularly safety, PROs, and economic criteria.

### 3.6. Final Meeting

A final meeting was held with the SC to perform a qualitative evaluation of the study results. This meeting was used to assess the plausibility and consistency of the quantitative findings, clarify areas of uncertainty, and contextualize the results according to clinical practice and healthcare decision-making in Spain.

#### 3.6.1. Weighting of Criteria

With regard to the weighting established during the assessment, the SC agreed that efficacy and safety parameters typically receive the highest scores, while the remaining criteria tend to fall within expected ranges. They highlighted that the overall therapeutic benefit is largely captured within the efficacy domain and emphasized that variability in other medical costs does not constitute a key aspect of the assessment. With respect to the efficacy sub-criteria, the SC agreed that OS represents the parameter providing the greatest value, followed by PFS. In contrast, panel members assigned lower scores to the response rate and duration of response. Although relevant in an incurable disease, these outcomes were considered to involve greater uncertainty regarding long-term benefit. Concerning AEs, the SC noted that the finding whereby AEs leading to treatment discontinuation received lower scores than severe or very severe AEs may appear counterintuitive. However, this was attributed to the relatively low frequency of discontinuations due to AEs with the therapies evaluated, as well as to risk–benefit considerations inherent in the treatment of a serious disease such as EC. In this context, the SC considered that continuing an effective treatment is often preferable to discontinuation due to AEs. Finally, the experts expressed some disagreement regarding the weighting assigned to PROs, as adherence and HRQoL received similar scores. Given that most of the evaluated treatments are administered intravenously, the SC considered that adherence played a less critical role in this context, whereas HRQoL was regarded as deserving greater relative importance.

#### 3.6.2. Scoring of Criteria

With regard to the evidence matrix, particularly in the comparison between dostarlimab and durvalumab/durvalumab + olaparib, differences emerged between the perspectives of oncologists and hospital pharmacists. Oncologists primarily focused on clinical trial design and efficacy outcomes, highlighting the OS benefit observed with dostarlimab and the comparatively less mature safety data available for durvalumab. In contrast, hospital pharmacists emphasized adherence to reference clinical guidelines, in which both treatments are similarly positioned. Nevertheless, both groups acknowledged that dostarlimab may offer more favorable efficacy and safety outcomes overall. No additional comments were provided by the SC regarding the remaining sub-criteria. Regarding the comparison with pembrolizumab, the experts perceived a consistent trend in favor of dostarlimab in terms of both efficacy and safety. This assessment was supported by the robustness of the available data and by the characteristics of the patient populations included in the clinical trials, which also encompassed patients beyond those with endometrial histology. Hospital pharmacists noted that, although the frequency and profile of toxicities may differ between treatments, the overall impact on HRQoL appears to be comparable, and emphasized the need for further evidence on the long-term use of pembrolizumab.

The SC also expressed disagreement with the cost-related findings, as these were derived exclusively from publicly available information and did not account for confidential pricing or funding agreements.

#### 3.6.3. Scoring of Sub-Criteria

Regarding the comparative sub-criteria, the SC considered that the analysis against pembrolizumab more accurately reflects clinical reality, particularly by clearly highlighting OS as the most relevant outcome. The remaining parameters were considered to fall within a similar range, although with a consistent trend in favor of dostarlimab, likely attributable to its longer follow-up duration (approximately 3 years).

#### 3.6.4. Scoring of Contextual Criteria

Regarding the contextual criteria, the SC emphasized the importance of interpreting these results in light of the qualitative judgment provided by the SC, given the inherent complexity of these issues. They highlighted that contextual criteria often require a nuanced interpretation that goes beyond mere quantitative scoring.

#### 3.6.5. Value Contribution

Finally, with respect to the overall value contribution, the SC agreed that the results were coherent and consistent with their clinical experience and expectations, and no additional comments or modifications were proposed.

## 4. Discussion

The present study aimed to assess the value contribution of dostarlimab in the first-line treatment of adult patients with advanced or recurrent primary EC who are candidates for systemic therapy in Spain. To our knowledge, this is the first MCDA applied in the field of EC in Spain and the first MCDA that estimates the value of a treatment for EC.

MCDA has been increasingly recognized as a suitable methodology for comprehensively capturing the multidimensional value of healthcare interventions [21,23,24]. Its growing popularity in recent years reflects its ability to integrate, in a transparent and structured manner, all the attributes associated with therapeutic alternatives across multiple disease areas. This holistic perspective is particularly relevant in settings characterized by complexity and uncertainty, where traditional single-criterion approaches may be insufficient. The MCDA approach is intended to complement, rather than replace, conventional health technology assessment methodologies, and its results should therefore be interpreted as reflecting the structured preferences of a multidisciplinary panel within a defined context [25]. Indeed, MCDA has frequently been applied in the evaluation of orphan drugs [26,27] and has shown increasing applicability within the Spanish healthcare context [28,29], with the EVIDEM framework being the most widely employed [30,31]. From a health technology assessment (HTA) perspective, MCDA has emerged as a valuable tool to support, rather than dictate, decision-making processes. A recent systematic review reported that HTA bodies have employed MCDA approaches in up to 40% of studies related to drug pricing decisions [32,33], underscoring its relevance in contemporary healthcare decision-making.

The use of MCDA has been particularly prominent in oncology [34,35,36], which represents the most extensively studied therapeutic area using this methodology [37]. This is unsurprising, given the high unmet medical need, the complexity of treatment pathways, the relevance of survival outcomes, and the substantial toxicity and economic burden associated with cancer therapies. Previous authors have emphasized the importance of conducting MCDA at a regional level to adequately reflect contextual and healthcare system-specific factors influencing value perception [38].

In line with methodological recommendations, especially for oncology-focused MCDA studies, the present work incorporated a multidisciplinary panel including oncologists and hospital pharmacists [39,40], and involved a substantial number of experts, as recommended in prior publications [41,42,43]. Furthermore, the hierarchical weighting strategy and value measurement approach employed in this study are consistent with those most frequently used in oncology MCDA research [33,44,45] and align with ISPOR methodological recommendations [44]. These elements collectively strengthen the robustness, transparency, and contextual relevance of the study design.

Overall, the MCDA results indicate that dostarlimab showed a positive added value in both comparisons in the first-line treatment setting, with total value contributions of +0.58 relative to durvalumab/durvalumab plus olaparib and +0.50 relative to pembrolizumab. These values fall within the range observed in other analyses employing the EVIDEM framework [22,31,41,46,47,48,49,50]. Nevertheless, these results should be interpreted in light of differences in the maturity and availability of evidence across comparators.

It is important to highlight that dostarlimab and pembrolizumab are currently the treatments funded by the Spanish National Health System [51]. Therefore, the comparison with pembrolizumab is particularly relevant in the context of approved and funded treatments in Spain.

Consistent with previous oncology-focused MCDA studies, the criteria contributing most substantially to the overall value were those related to disease severity, treatment efficacy, and safety, clearly outweighing cost-related criteria [42]. This pattern reflects the clinical reality of decision-making in oncology, where outcomes such as OS and PFS, as well as the toxicity profile of treatments, are prioritized over economic considerations [30,52]. In this context, the significant contribution of the quality of evidence criterion observed in our study is also in line with recent publications highlighting the importance of robust and mature clinical data in value assessment exercises [53,54,55].

The contribution of efficacy-related criteria is supported by the available evidence indicating that dostarlimab may have favorable effects on OS and PFS compared with pembrolizumab [56,57]. Although pembrolizumab may show higher PFS and duration of response in specific subgroups, the available evidence for dostarlimab includes statistically significant OS in the overall population as the primary endpoint and more mature evidence compared to pembrolizumab. These findings are also consistent with recent economic evaluations in which dostarlimab has been identified as a cost-effective option compared with pembrolizumab or the durvalumab-plus-olaparib combination, particularly in the dMMR/MSI-H and pMMR/MSS populations [58,59]. Overall, the consistency between the MCDA results and the clinical and economic literature reinforces the credibility of the value estimates generated in this study.

From an economic perspective, the analysis has limitations as it was conducted using publicly available economic data without taking into account potential pricing and reimbursement agreements at the national or regional level. Although the results favored dostarlimab, qualitative findings suggested that pembrolizumab might offer advantages under such agreements that were not considered. Since the completion of this work and prior to its publication, the situation regarding pembrolizumab in Spain has changed [60]. Therefore, while the economic analysis remains relevant, the cost of treatments is an uncertain factor, and as such, there are certain limitations to the results of this criterion. Despite the strengths of the MCDA methodology in capturing the multidimensional value of therapeutic interventions, several limitations inherent to this approach apply to the present study. Firstly, the EVIDEM framework is based on a predefined set of criteria, which, while comprehensive, may not fully capture all potentially relevant dimensions of value in specific clinical contexts. In addition, MCDA processes are subject to inherent cognitive complexity and variability arising from expert deliberation, which has been widely reported in the literature [41,44,61]. This includes potential biases related to the subjective selection and weighting of criteria, particularly when hierarchical weighting strategies are used. Although qualitative criteria were reported to contextualize ethical and subjective aspects of value, these elements are inherently difficult to quantify and cannot be fully integrated into the calculation of the total value contribution. Sensitivity analyses using alternative weighting scenarios were not performed. Although such analyses may be useful to explore the robustness of MCDA findings, they were not prespecified in the present study, which was designed as a single structured elicitation exercise based on the preferences of the participating expert panel. Future MCDA studies in this setting could prospectively incorporate sensitivity analyses to assess the impact of alternative weighting assumptions on the overall value contribution. The focus of the study, restricted to the Spanish healthcare system, represents another limitation, as it may limit the extrapolation of the findings to other settings, despite recommendations supporting region-specific MCDA analyses. While previous authors have recommended including patient perspectives [40,62,63], putting this into practice remains challenging due to the lack of standardized methodologies. Although patient input could not be included in this study, the expert panel was highly robust, consisting of 12 oncologists and 13 HP representing 11 of the 17 ACs, ensuring a broad and meaningful professional perspective.

Finally, the contextualization of certain criteria relied exclusively on publicly available information, as there was no access to confidential data, such as negotiated drug prices or future financial agreements. The absence of head-to-head clinical trials and reliance on price estimates derived from other indications further limit the accuracy of some comparisons. Future studies incorporating more mature clinical data and access to confidential economic information could therefore provide additional value and refine the conclusions of the present work.

The potential influence of sponsorship represents an important consideration when interpreting the results of this study. Although the sponsor was involved in the funding of the research, the selection of criteria, weighting, and scoring processes were conducted independently by a multidisciplinary panel of external experts, following internationally recognized methodological standards (ISPOR and EVIDEM). No individual-level scores were modified or adjusted by the sponsor, and all value estimates reflect the aggregated judgments of the participating clinicians and hospital pharmacists.

## 5. Conclusions

The results obtained through a structured MCDA suggest that dostarlimab is a treatment option that offers a higher overall value contribution for adult patients with advanced or recurrent primary EC, particularly in terms of OS, compared to other conventional alternatives. This finding was observed across the two treatment comparisons evaluated and reflects the integration of available clinical evidence, expert judgement, and contextual considerations within the Spanish healthcare setting. Pembrolizumab and dostarlimab are currently the only options available to patients under the Spanish public healthcare system; therefore, the comparison between these two treatments is particularly relevant within the current Spanish healthcare context [51]. In terms of efficacy, dostarlimab has demonstrated a statistically significant OS as the primary endpoint, providing an overall benefit in the overall population and more robust clinical evidence. In addition, available results suggest potentially more favorable long-term quality-of-life outcomes and a manageable toxicity profile, which could promote treatment continuity and adherence in clinical practice compared to pembrolizumab. Based on the available evidence, the expert panel considered these attributes to favor dostarlimab over pembrolizumab. Nevertheless, these comparative findings should be interpreted with caution, as they are not based on direct head-to-head evidence between dostarlimab and the comparators, but rather on a structured assessment of the available evidence and expert scoring.

The assessment of contextual criteria further indicates that dostarlimab is generally well aligned with the priorities of the national healthcare system, particularly regarding access to innovative therapies for vulnerable patient populations. The expert-perceived improvements in efficacy, HRQoL, and survival, together with the potential for long-term optimization of healthcare resource use, support the consideration of dostarlimab as a valuable therapeutic option in the management of first-line treatment for adult patients with advanced or recurrent primary EC who are candidates for systemic therapy in Spain. The MCDA methodology has played a crucial role in this study, as it provides a systematic, transparent, and multidimensional framework to support decision-making, enabling a comprehensive evaluation of healthcare technologies based on various criteria beyond clinical efficacy alone.

## Figures and Tables

**Table 1:** Scores assigned to each criterion and comments from the expert panel.

Criteria	Scores	Comments
Disease severity	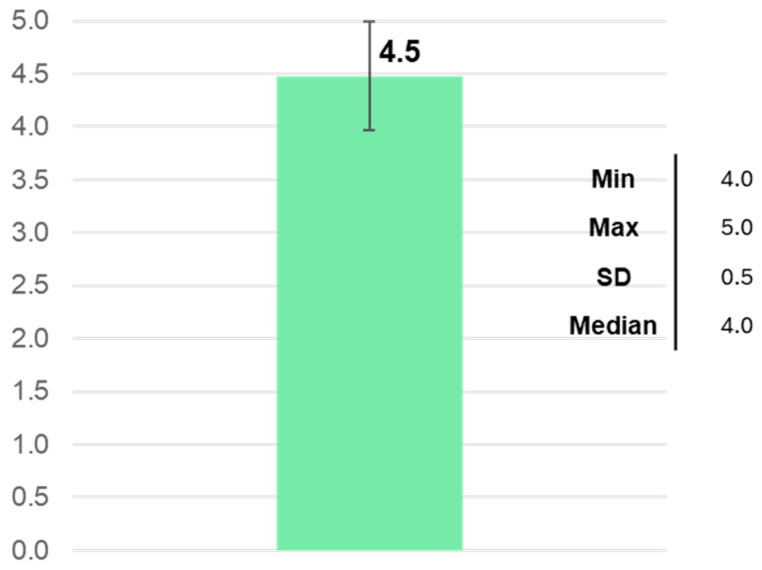	- The expert panel agreed that advanced or recurrent EC is a very severe disease, primarily due to its poor prognosis, low survival rates, and limited treatment options. In addition, this condition mainly affects older and often frail women, further worsening clinical outcomes and overall prognosis. - The expert panel considered that the disease has a profound impact on patients’ quality of life.
Size of the affected population	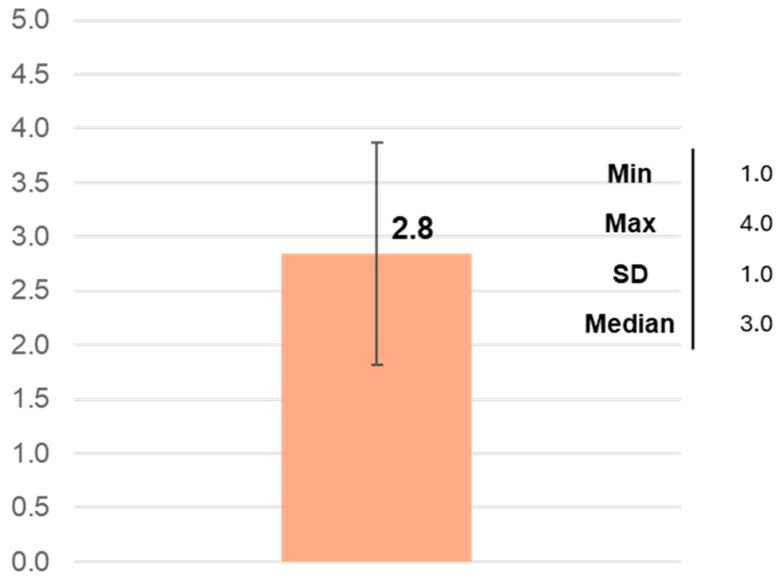	- The expert panel agreed that only a small proportion of EC cases are diagnosed at advanced stages or present as recurrent disease. Consequently, they considered the affected population to be relatively small, despite the observed increase in prevalence. - Several experts attributed this increase to rising rates of obesity and sedentary lifestyles, as well as to improved survival rates associated with the availability of more effective treatments.
Unmet needs	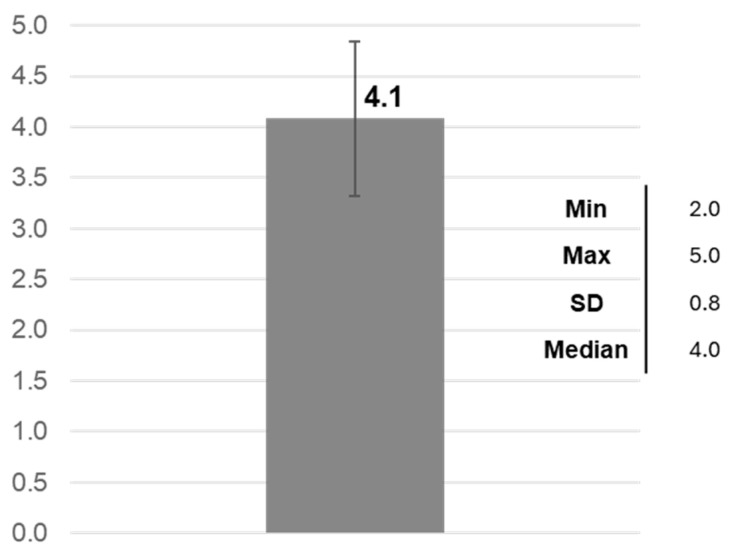	- The panel agreed that, despite recent advances in molecular classification and the emergence of novel therapies, access to comprehensive molecular testing remains uneven. This limitation hampers the individualized selection of optimal therapeutic strategies and restricts the full implementation of precision medicine approaches. - While experts acknowledged the significant progress made with immunotherapy, they emphasized that, despite offering durable efficacy for some patients, these treatments continue to pose specific clinical and management challenges. - The panel highlighted the ongoing need to improve patients’ quality of life, which is substantially affected by both the disease itself and treatment-related adverse events. - Experts also noted that clinical research in this setting remains limited and that EC lacks clearly defined therapeutic goals in advanced or recurrent stages, which constrains the development and evaluation of more effective treatment options.
Efficacy	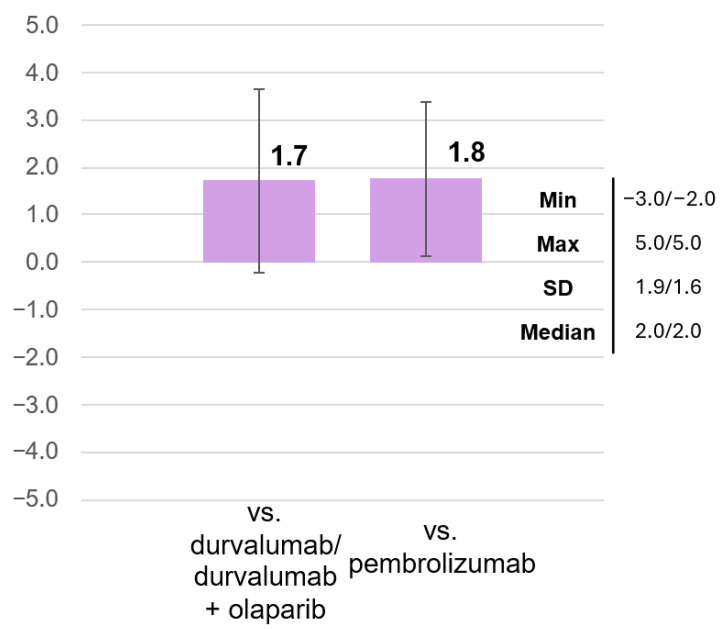	Dostarlimab vs. durvalumab/durvalumab + olaparib - Dostarlimab was associated with improved OS and duration of response, particularly in patients with dMMR/MSI-H tumors. In contrast, the combination of durvalumab plus olaparib showed promising PFS results in the pMMR/MSS population and may represent a potential option for selected subgroups, albeit at the expense of increased toxicity. - A key advantage of dostarlimab lies in the longer follow-up period and the robustness and consistency of the results observed in the RUBY trial, especially regarding OS outcomes. Dostarlimab vs. pembrolizumab - The expert panel considered that the available evidence for dostarlimab suggested a favorable OS profile and greater robustness of evidence, supported by a longer follow-up period. However, in terms of PFS and duration of response, pembrolizumab may show numerically higher outcomes in specific subgroups, although additional data and longer follow-up are required to confirm these findings. - In the dMMR/MSI-H population, dostarlimab appears to provide a greater therapeutic benefit, whereas in the pMMR/MSS population, pembrolizumab may offer slightly superior results for specific efficacy parameters, such as PFS.
Safety	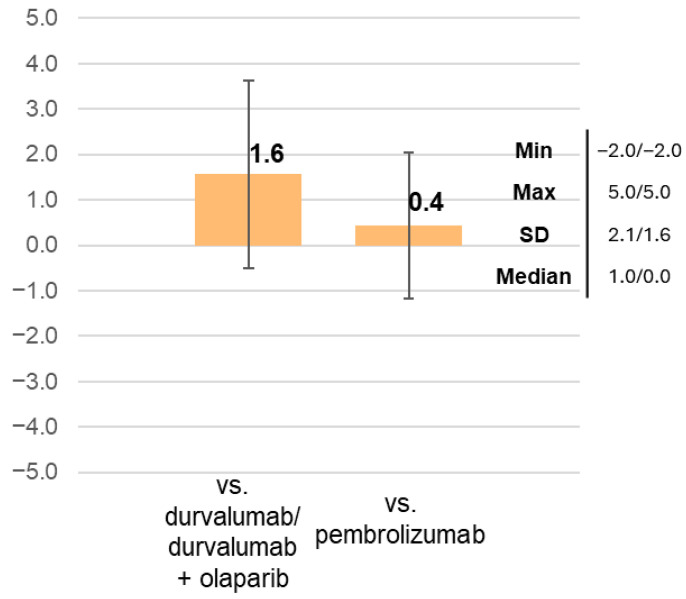	Dostarlimab vs. durvalumab/durvalumab + olaparib - Both treatments were considered to have overall manageable safety profiles. However, dostarlimab, when administered as monotherapy, was associated with a more predictable toxicity profile. Although a higher incidence of very severe AEs was reported with dostarlimab, it exhibited a more favorable profile in terms of very common AEs, which positively influenced the panel’s overall assessment of the RUBY study results. - In contrast, the addition of olaparib was associated with an increased toxicity burden, particularly hematological adverse events such as anemia and thrombocytopenia, as well as higher rates of permanent treatment discontinuation. Dostarlimab vs. pembrolizumab - Most experts agreed that both treatments present broadly comparable toxicity profiles. However, dostarlimab was considered to have the advantage of a longer follow-up period and a more established adverse event reporting system, which enhances the robustness and consistency of the available safety data. Some experts perceived dostarlimab as potentially better tolerated, with fewer severe adverse events and lower rates of treatment discontinuation.
PROs	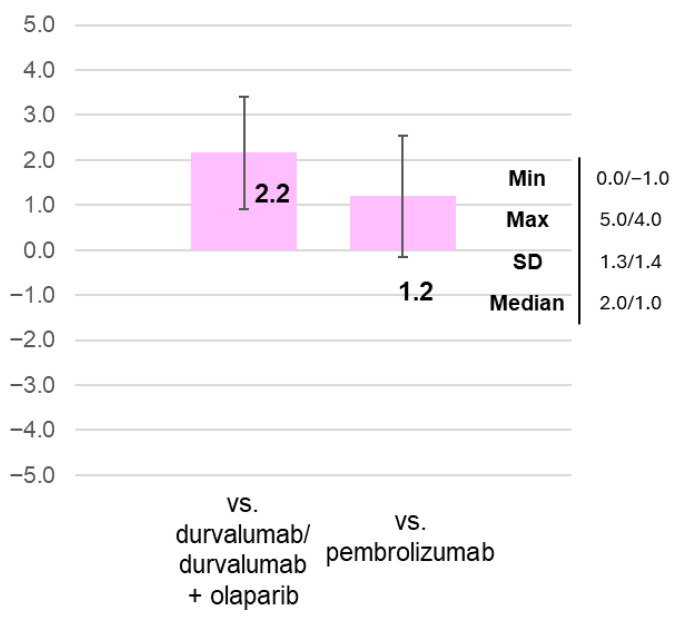	Dostarlimab vs. durvalumab/durvalumab + olaparib - Based on expert panel assessment of patient-reported outcome data, the dostarlimab regimen is perceived as more advantageous than that of durvalumab. This benefit appears to outweigh the requirement for a daily oral treatment in combination regimens, as dostarlimab may improve adherence, reduce the therapeutic burden, minimize administration errors, and better preserve patients’ quality of life. In contrast, the addition of olaparib to the treatment regimen increases toxicity, negatively affects quality of life, and is associated with a higher risk of adherence issues. Overall, dostarlimab is perceived as a more convenient and better-tolerated option, with a more favorable quality-of-life profile for patients. Dostarlimab vs. pembrolizumab - Dostarlimab appears to provide more favorable outcomes in terms of long-term quality-of-life, particularly in the dMMR/MSI-H population. Nevertheless, its longer treatment duration may represent a disadvantage for some patients in terms of comfort and adherence. Conversely, pembrolizumab offers the advantage of a shorter treatment duration; however, this does not translate into an improvement in patients’ quality of life. Notably, the reported quality-of-life outcomes for pembrolizumab derive from the pMMR population and indicate a slight deterioration in this parameter.
Type of therapeutic benefit	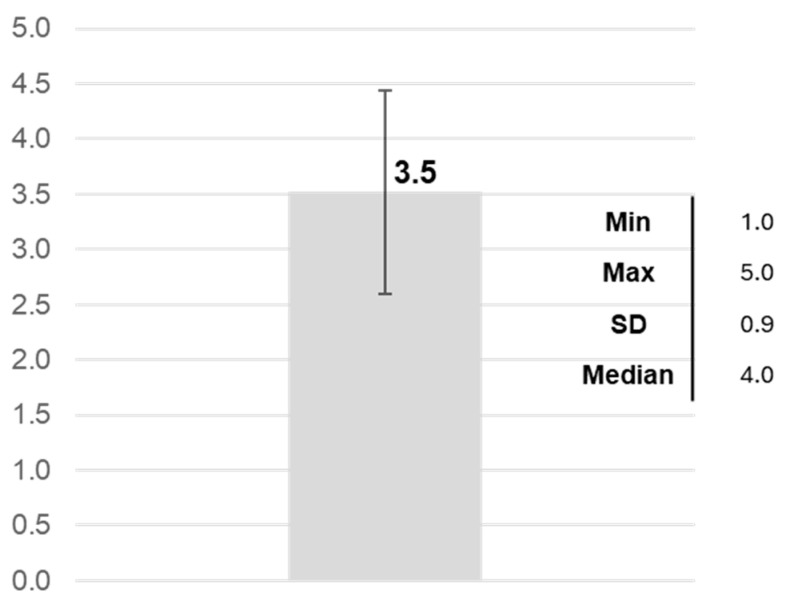	- The experts agreed that immunotherapy in this setting is not a curative treatment; however, dostarlimab provides clinically meaningful benefits in terms of survival and quality of life. In particular, the panel highlights its favorable impact on PFS and OS, especially in patients with dMMR/MSI-H tumors, who tend to exhibit more durable responses to treatment. - With regard to quality of life, dostarlimab is considered to have a positive effect, as it maintains and, in some cases, improves disease-related symptoms without imposing a substantial additional toxicity burden. However, some experts note that the magnitude of the benefit appears to be more limited in the pMMR/MSS population.
Cost of intervention	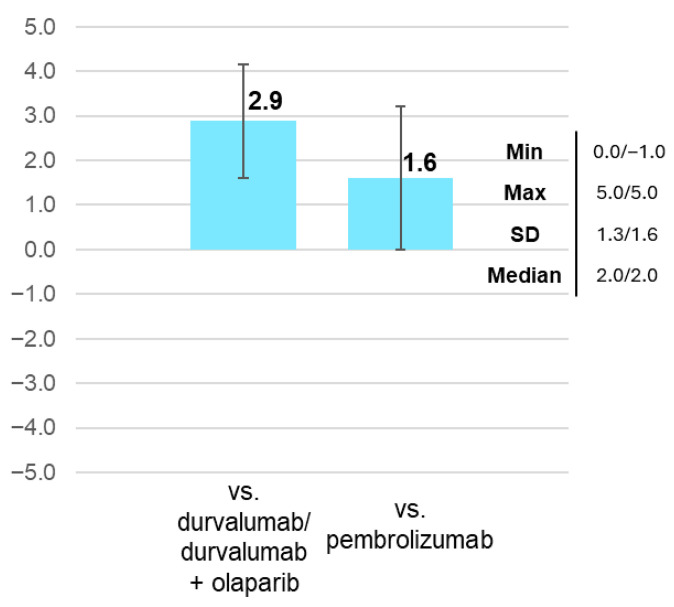	Dostarlimab vs. durvalumab/durvalumab + olaparib - The panel considers dostarlimab to be a more economical option than durvalumab and the durvalumab–olaparib combination, both in terms of direct pharmacological costs and indirect healthcare-related costs. - Its use as intravenous monotherapy, together with a predefined maximum treatment duration of three years, contributes to a more cost-efficient approach, particularly when compared with combination regimens that continue until disease progression or unacceptable toxicity. - Although individual costs may vary depending on local pricing, reimbursement conditions, and funding agreements, experts generally agree that dostarlimab represents a more economical and efficient option for the healthcare system. Dostarlimab vs. pembrolizumab - Overall, dostarlimab is generally considered a more cost-effective option than pembrolizumab, primarily due to its lower annual pharmacological cost. However, variability in pembrolizumab dosing (including weight-based adjustments) and differences in pricing and reimbursement agreements at national or regional levels could diminish the cost-effectiveness advantage of dostarlimab.
Other medical costs	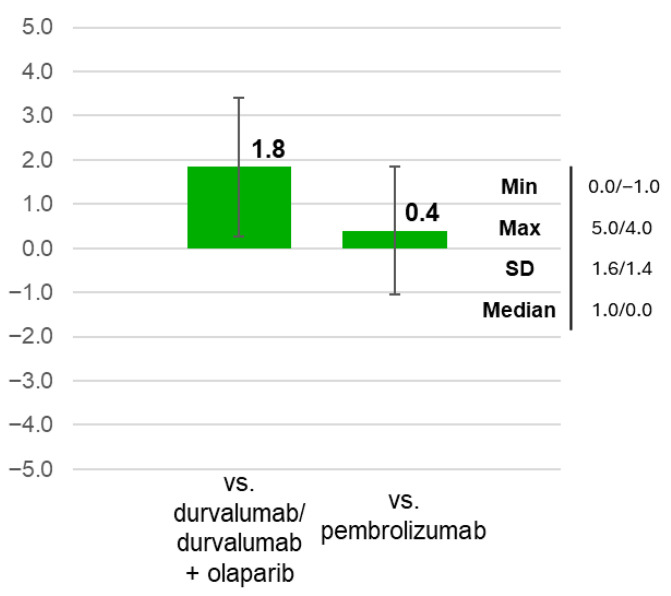	Dostarlimab vs. durvalumab/durvalumab + olaparib - Dostarlimab and durvalumab have comparable costs when used as monotherapy. However, when durvalumab is combined with olaparib, overall costs increase substantially. This increase is driven by the higher toxicity profile of olaparib, which results in more hospitalizations, additional supportive treatments, and higher pharmacy-related costs, including those associated with outpatient pharmaceutical consultations. Dostarlimab vs. pembrolizumab - The experts did not identify substantial differences between the two treatments in terms of overall cost impact. Pembrolizumab is administered over a shorter treatment period, which could limit long-term expenditure, while dostarlimab may offer an advantage due to its lower toxicity profile, potentially translating into reduced hospitalization and management costs; this perception may be supported by the accumulated experience with dostarlimab in the treatment of endometrial cancer in a first-line setting.
Quality of evidence	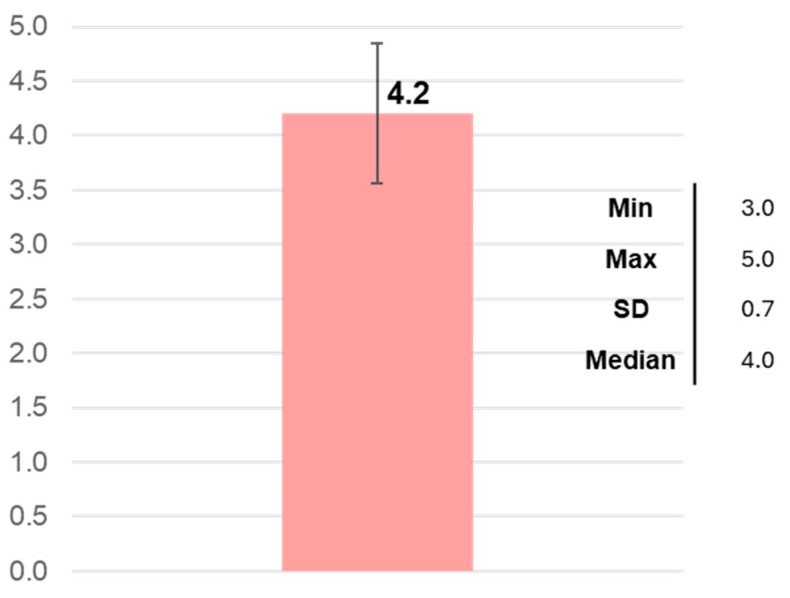	- Most experts consider that the available evidence in favor of dostarlimab is strong or moderately strong. This assessment is supported by an ESMO-MCBS score of 4 and a high GRADE level, both being key indicators of the robustness and reliability of the evidence. - The panel particularly valued the data from the RUBY study due to its rigorous methodological design, which included randomization, double-blind conditions, and a comparator group. In addition, the study provided evidence supporting clinically meaningful improvements in both PFS and OS as primary endpoint, particularly in the dMMR/MSI-H population, which led the expert panel to assign a high degree of confidence in the strength of the evidence. - Some experts noted that, in the pMMR/MSS population, the magnitude of benefit appears more modest. However, the majority of the panel agreed that dostarlimab remains an appropriate and valuable therapeutic option for this patient subgroup.
Expert consensus/CPG	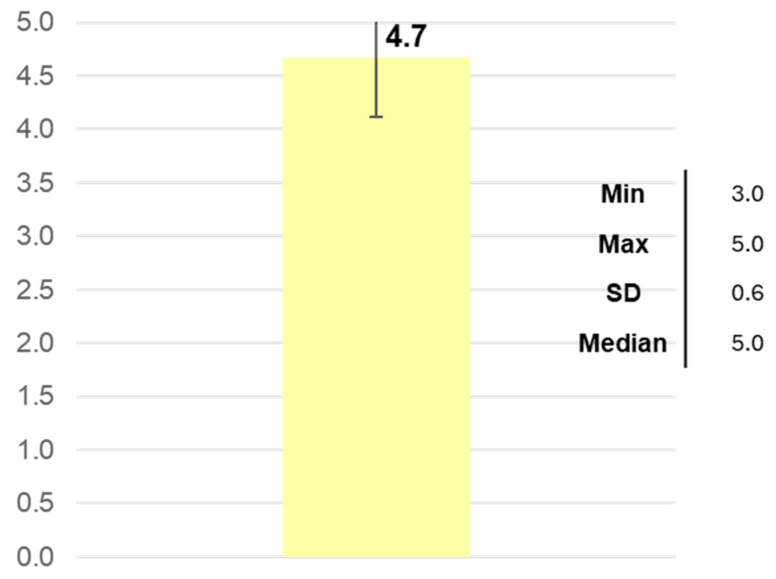	- Dostarlimab is strongly supported by leading national and international clinical practice guidelines, with high-quality evidence (Grade I). The strength of the recommendation varies according to the patient population: a grade 1A recommendation, based on robust evidence, for the dMMR/MSI-H population, and a grade 1B recommendation, based on moderate evidence, for the pMMR/MSS population.

AE, adverse event; ESMO, European Society of Medical Oncology; GRADE, Grading of Recommendations, Assessment, Development and Evaluation; MCBS, magnitude of clinical benefit scale; MSI-H, microsatellite Instability-High; MSS, microsatellite stable; OS, overall survival; PFS, progression-free survival; pMMR, proficient mismatch repair; QoL, quality of life; SD, standard deviation Among the comparative criteria, values on the left (min, max, SD, and median) represent the comparison with durvalumab/durvalumab + olaparib, whereas values shown on the right refer to the comparison with pembrolizumab.

## Data Availability

All data presented in this study are available on the Supplementary Materials. Further inquiries can be directed to the corresponding author.

## Supplementary Materials

The following supporting information can be downloaded at: https://www.mdpi.com/article/10.3390/jcm15155808/s1, Table S1: Search strategy; Table S2: Included and excluded criteria.

## Abbreviations

The following abbreviations are used in this manuscript:

ACs	Autonomous communities
AE	Adverse event
CPG	Clinical practice guidelines
CSI	Critical & Sensitive Information
dMMR	Deficient mismatch repair
dMMR/MSI-H	Deficient mismatch repair/microsatellite instability-high
EC	Endometrial cancer
EC-MCDA	Endometrial cancer multi-criteria decision analysis
ESMO	European Society of Medical Oncology
ESMO-MCBS	European Society of Medical Oncology Magnitude of Clinical Benefit Scale
EVIDEM	Evidence and Value: Impact on Decision Making
GRADE	Grading of Recommendations, Assessment, Development and Evaluation
HP	Hospital pharmacist(s)
HRQoL	Health-related quality of life
HTA	Health technology assessment
ISPOR	International Society for Pharmacoeconomics and Outcomes Research
MCDA	Multi-criteria decision analysis/multicriteria decision analysis
MeSH	Medical Subject Headings
MMR	Mismatch repair
MSI	Microsatellite instability
MSI-H	Microsatellite instability-high
MSI-L	Microsatellite instability-low
MSS	Microsatellite stable/microsatellite stability
OS	Overall survival
PFS	Progression-free survival
pMMR	Proficient mismatch repair
pMMR/MSS	Proficient mismatch repair/microsatellite stable
PROs	Patient-reported outcomes
QoL	Quality of life
SC	Scientific Committee
SD	Standard deviation
SEOM	Spanish Society of Medical Oncology
Vx	Value contribution of each quantitative criterion
Wx	Normalized weight
Sx	Standardized score
