# Supplementary material for: The Contribution of Dostarlimab to First-Line Treatment of Advanced or Recurrent Endometrial Cancer in Spain: A Multi-Criteria Decision Analysis Approach
Source: J Clin Med. 2026 Jul 24;15(15):5808. doi: 10.3390/jcm15155808 (PMC13467585; doi:10.3390/jcm15155808)
Supplement: Supplementary file 1 [file jcm-15-05808-s001.zip › jcm-4382315-supplementary.pdf]

Supplementary Table S1 Search strategy

	DISEASE CRITERIA
	**Disease-related terms**
#1	"advanced endometrial neoplasms" [Mesh]
	**Treatment-related terms**
#2	" dostarlimab" [Title/Abstract]
#3	" durvalumab" [Title/Abstract]
#4	" pembrolizumab" [Title/Abstract]
	((#1) AND (#2 OR #3 OR #4)) ("endometrial neoplasms"[MeSH Terms] AND ("dostarlimab"[Title/Abstract] OR "durvalumab"[Title/Abstract] OR "pembrolizumab"[Title/Abstract])) AND ((y_10[Filter]) AND (clinicaltrial[Filter] OR clinicaltrialphaseiii[Filter] OR clinicaltrialphaseiv[Filter] OR guideline[Filter] OR meta-analysis[Filter] OR observationalstudy[Filter] OR systematicreview[Filter]) AND (humans[Filter]) AND (english[Filter] OR spanish[Filter]))
	**Disease severity-related terms**
#5	"disease severity" [Title/Abstract]
#6	"severity" [Title/Abstract]
#7	"morbidity" [Title/Abstract]
#8	"life-expectancy" [Title/Abstract]
#9	"burden" [Title/Abstract]
	((#1) AND (#5 OR #6 OR #7 OR #8 OR #9)) ("endometrial neoplasms"[MeSH Terms] AND ("disease severity"[Title/Abstract] OR "severity"[Title/Abstract] OR "morbidity"[Title/Abstract] OR "life-expectancy"[Title/Abstract] OR "burden"[Title/Abstract])) AND ((y_10[Filter]) AND (clinicaltrial[Filter] OR clinicaltrialphaseiii[Filter] OR clinicaltrialphaseiv[Filter] OR guideline[Filter] OR meta-analysis[Filter] OR observationalstudy[Filter] OR systematicreview[Filter]) AND (humans[Filter]) AND (english[Filter] OR spanish[Filter]))
	**Epidemiology-related terms**
#10	"incidence" [Title/Abstract]
#11	"prevalence" [Title/Abstract]
#12	"europe" [Title/Abstract]
#13	"european" [Title/Abstract]
#14	"spain" [Title/Abstract]
#15	"spanish" [Title/Abstract]
	(#1 AND (#10 OR #11) AND (#12 OR #13 OR #14 OR #15)) ("endometrial neoplasms"[MeSH Terms] AND ("incidence"[Title/Abstract] OR "prevalence"[Title/Abstract]) AND ("europe"[Title/Abstract] OR "european"[Title/Abstract] OR "spain"[Title/Abstract] OR "spanish"[Title/Abstract])) AND ((y_10[Filter]) AND (clinicaltrial[Filter] OR clinicaltrialphaseiii[Filter] OR clinicaltrialphaseiv[Filter] OR guideline[Filter] OR meta-analysis[Filter] OR observationalstudy[Filter] OR systematicreview[Filter]) AND (humans[Filter]) AND (english[Filter] OR spanish[Filter]))
	**Unmet needs-related terms**
#16	"unmet need" [Title/Abstract]
#17	"patient need" [Title/Abstract]
#18	"caregiver need" [Title/Abstract]
	((#1) AND (#16 OR #17 OR #18))

CONFIDENCIAL

	(("endometrial neoplasms"[MeSH Terms] AND ("unmet need"[Title/Abstract] OR "patient need"[Title/Abstract] OR "caregiver need"[Title/Abstract])) AND ((y_10[Filter]) AND (clinicaltrial[Filter] OR clinicaltrialphaseiii[Filter] OR clinicaltrialphaseiv[Filter] OR guideline[Filter] OR meta-analysis[Filter] OR observationalstudy[Filter] OR systematicreview[Filter]) AND (humans[Filter]) AND (english[Filter] OR spanish[Filter]))
	**INTERVENTION CRITERIA**
	**Efficacy-related terms**
#19	"efficacy" [Mesh]
#20	"progression-free survival" [Title/Abstract]
#21	"overall survival" [Title/Abstract]
	((#1) AND (#2 OR #3 OR #4) AND (#19 OR #20 OR #21)) ("endometrial neoplasms"[MeSH Terms] AND ("dostarlimab"[Title/Abstract] OR "durvalumab"[Title/Abstract] OR "pembrolizumab"[Title/Abstract]) AND ("progression free survival"[Title/Abstract] OR "overall survival"[Title/Abstract])) AND ((y_10[Filter]) AND (clinicaltrial[Filter] OR clinicaltrialphaseiii[Filter] OR clinicaltrialphaseiv[Filter] OR guideline[Filter] OR meta-analysis[Filter] OR observationalstudy[Filter] OR systematicreview[Filter]) AND (humans[Filter]) AND (english[Filter] OR spanish[Filter]))
	**Safety-related terms**
#22	"safety" [Mesh]
#23	"adverse events" [Title/Abstract]
#24	"interruption" [Title/Abstract]
#25	"discontinuation" [Title/Abstract]
	((#1) AND (#2 OR #3 OR #4) AND (#22 OR #23 OR #24 OR #25)) (("endometrial neoplasms"[MeSH Terms] AND ("dostarlimab"[Title/Abstract] OR "durvalumab"[Title/Abstract] OR "pembrolizumab"[Title/Abstract]) AND ("safety"[MeSH Terms] OR "adverse events"[Title/Abstract] OR "interruption" [Title/Abstract] OR "discontinuation" [Title/Abstract])) AND ((y_10[Filter]) AND (clinicaltrial[Filter] OR clinicaltrialphaseiii[Filter] OR clinicaltrialphaseiv[Filter] OR guideline[Filter] OR meta-analysis[Filter] OR observationalstudy[Filter] OR systematicreview[Filter]) AND (humans[Filter]) AND (english[Filter] OR spanish[Filter]))
	**PROs-related terms**
#26	"quality of life" [Title/Abstract]
#27	"patient reported outcomes" [Title/Abstract]
#28	"convenience" [Title/Abstract]
	((#1) AND (#2 OR #3 OR #4) AND (#26 OR #27 OR #28)) ("endometrial neoplasms"[MeSH Terms] AND ("dostarlimab"[Title/Abstract] OR "durvalumab"[Title/Abstract] OR "pembrolizumab"[Title/Abstract]) AND ("quality of life"[Title/Abstract] OR "patient reported outcomes"[Title/Abstract] OR "convenience"[Title/Abstract])) AND ((y_10[Filter]) AND (clinicaltrial[Filter] OR clinicaltrialphaseiii[Filter] OR clinicaltrialphaseiv[Filter] OR guideline[Filter] OR meta-analysis[Filter] OR observationalstudy[Filter] OR systematicreview[Filter]) AND (humans[Filter]) AND (english[Filter] OR spanish[Filter]))
	**Therapeutic benefit-related terms**
#29	"treatment outcome" [Title/Abstract]
#30	"therapeutic benefit" [Title/Abstract]
#31	"clinical benefit" [Title/Abstract]
	((#2 OR #3 OR #4) AND (#29 OR #30 OR #31)) (("dostarlimab"[Title/Abstract] OR "durvalumab"[Title/Abstract] OR "pembrolizumab"[Title/Abstract]) AND ("treatment outcome"[Title/Abstract] OR

CONFIDENTIAL

	"therapeutic benefit"[Title/Abstract] OR "clinical benefit"[Title/Abstract])) AND ((y_10[Filter]) AND (clinicaltrial[Filter] OR clinicaltrialphaseiii[Filter] OR clinicaltrialphaseiv[Filter] OR guideline[Filter] OR meta-analysis[Filter] OR observationalstudy[Filter] OR systematicreview[Filter]) AND (humans[Filter]) AND (english[Filter] OR spanish[Filter]))
	**Costs-related terms**
#32	"disease cost" [All Fields]
#33	"medical costs" [All Fields]
#34	"non medical costs" [All Fields]
#35	"cost of illness" [All Fields]
	((#1) AND (#32 OR #33 OR #34 OR #35)) ("endometrial neoplasms"[MeSH Terms] AND ("disease cost"[All Fields] OR "medical cost"[All Fields] OR "non medical costs"[All Fields] OR "cost of illness"[All Fields]) AND ((y_10[Filter]) AND (humans[Filter]) AND (english[Filter] OR spanish[Filter]))
	**Knowledge of the intervention-related terms**
#36	"guideline" [Title/Abstract]
#37	"clinical practice" [Title/Abstract]
	((#1) AND (#36 OR #37)) ("endometrial neoplasms"[MeSH Terms] AND ("guideline"[Title/Abstract] OR "clinical practice"[Title/Abstract]) AND ((y_10[Filter]) AND (guideline[Filter]) AND (humans[Filter]) AND (english[Filter] OR spanish[Filter]))

Supplementary Table S2 Included and excluded criteria

	**Quantitative criteria**	**Sub-criteria**
**INCLUDED**	Disease severity	• Disease impact on morbidity<br>• Disease impact on QoL of patients<br>• Disease impact on life expectancy
	Size of the affected population	• Mean age<br>• Prevalence<br>• Incidence
	Unmet needs	• Unmet need for treatment
	Efficacy	• Overall survival (OS)<br>• Progression-free survival (PFS)<br>• Objective response rate (ORR)<br>• Response duration
	Safety	• Common and very common AEs<br>• Severe and very severe AEs<br>• AEs leading to discontinuation
	Patient Reported Outcomes (PROs)	• Convenience/treatment adherence<br>• HRQoL

CONFIDENTIAL

	Type of therapeutic benefit	• Cure of the disease<br>• Modification of the course of the disease<br>• Improvement of patients' QoL
	Cost of intervention	
	Other medical costs	• Administration costs<br>• Hospitalization cost due to an AE
	Quality of evidence	• ESMO-MCBS score<br>• GRADE
	Expert consensus/CPG	
	**Qualitative criteria**	**Sub-criteria**
	Mandate and scope of the health system	
	Population priorities and access	
	Common objective and specific interests	• Scientific societies and patient associations
	System capacity and appropriate use of intervention	• Institutional
	Opportunity costs and affordability	
**EXCLUDED**	**Quantitative criteria**	**Sub-criteria**
	Efficacy	• Progression-free survival 2
	Indirect costs of the treatment	• Travel costs<br>• Labor productivity costs
	**Qualitative criteria**	**Sub-criteria**
	Environmental impact	
	Historical/Political/Cultural context	

AE, adverse event; CPG, clinical practice guideline; ESMO, European society for medical oncology; GRADE, grading of recommendations assessment development and evaluation; HRQoL, health-related quality of life; MCBS, magnitude of clinical benefit scale; ORR, objective response rate; OS, overall survival; PFS, progression free survival; PRO, patient-reported outcome; QoL, quality of life

CONFIDENCIAL
